# DAPK loss in colon cancer tumor buds: implications for migration capacity of disseminating tumor cells

**DOI:** 10.18632/oncotarget.4908

**Published:** 2015-08-21

**Authors:** Jelena Ivanovska, Inti Zlobec, Stefan Forster, Eva Karamitopoulou, Heather Dawson, Viktor Hendrik Koelzer, Abbas Agaimy, Fabian Garreis, Stephan Söder, William Laqua, Alessandro Lugli, Arndt Hartmann, Tilman T. Rau, Regine Schneider-Stock

**Affiliations:** ^1^ Experimental Tumor Pathology, Institute of Pathology, Friedrich-Alexander University of Erlangen-Nürnberg (FAU), Erlangen, Germany; ^2^ Institute of Pathology, Friedrich-Alexander University of Erlangen-Nürnberg (FAU), Erlangen, Germany; ^3^ Institute of Pathology, University of Bern, Bern, Switzerland; ^4^ Department of Anatomy, Friedrich-Alexander University of Erlangen-Nürnberg (FAU), Erlangen, Germany

**Keywords:** DAPK, colorectal cancer, tumour budding, migration

## Abstract

Defining new therapeutic strategies to overcome therapy resistance due to tumor heterogeneity in colon cancer is challenging. One option is to explore the molecular profile of aggressive disseminating tumor cells. The cytoskeleton-associated Death-associated protein kinase (DAPK) is involved in the cross talk between tumor and immune cells at the invasion front of colorectal cancer. Here dedifferentiated tumor cells histologically defined as tumor budding are associated with a high risk of metastasis and poor prognosis. Analyzing samples from 144 colorectal cancer patients we investigated immunhistochemical DAPK expression in different tumor regions such as center, invasion front, and buds. Functional consequences for tumor aggressiveness were studied in a panel of colon tumor cell lines using different migration, wound healing, and invasion assays. DAPK levels were experimentally modified by siRNA transfection and overexpression as well as inhibitor treatments. We found that DAPK expression was reduced towards the invasion front and was nearly absent in tumor buds. Applying the ECIS system with HCT116 and HCT116 stable lentiviral DAPK knock down cells (HCTshDAPK) we identified an important role for DAPK in decreasing the migratory capacity whereas proliferation was not affected. Furthermore, the migration pattern differed with HCTshDAPK cells showing a cluster-like migration of tumor cell groups. DAPK inhibitor treatment revealed that the migration rate was independent of DAPK's catalytic activity. Modulation of DAPK expression level in SW480 and DLD1 colorectal cancer cells significantly influenced wound closure rate. DAPK seems to be a major player that influences the migratory capability of disseminating tumor cells and possibly affects the dynamic interface between pro- and anti-survival factors at the invasion front of colorectal cancer. This interesting and new finding requires further evaluation.

## INTRODUCTION

Death-associated protein kinase (DAPK) is a calmodulin regulated and cytoskeleton associated serine/threonine kinase [1]. It is a large 160 kDa protein composed of several functional domains, including a kinase domain, a CaM regulatory domain, eight consecutive ankyrin repeats, two putative nucleotide binding domains (P-loops), cytoskeletal/ras of complex proteins (ROC) domain and a death domain [2, 3]. Due to its multi-domain structure, DAPK's function is not only restricted to its kinase activity. Some studies report the importance of the scaffold function of DAPK to trigger and stabilise multi-protein complexes [4, 5].

Besides a major role in cell death mechanisms, DAPK also regulates cytoskeleton-associated proteins to suppress directed migration via blocking of cell polarisation [4, 6]. Kuo et al. [6] reported that DAPK has an inhibitory effect on talin-H association with integrins leading to an abrogation of their inside-out activation [7–9]. The phosphorylation of myosin light chain at Ser19 by DAPK *in vivo* correlates also with the weakening of the structural integrity of the cortical actin network necessary for morphological apoptosis-associated changes such as cell rounding, shrinking, and detachment [10–13].

Animal studies in syngenic mice have shown that lung carcinoma clones with highly aggressive metastatic behaviour do not express DAPK, in contrast to their low metastatic counterparts [14]. Moreover, DAPK interferes with both, early- and late-stage metastatic processes, suggesting that DAPK suppresses metastasis through multiple mechanisms [15]. In patients with colon cancer, the late DAPK down-regulation is associated with metastasis to lymph nodes and distant organs, as well as with a shorter metastasis-free period and reduced overall survival [15]. In line with the anti-metastatic function of DAPK, clinical studies indicate that loss of DAPK expression in several cancer types, by hypermethylation of the DAPK promoter, is associated with advanced tumor stages and more aggressive phenotypes [15, 16]. DAPK overexpressing uterine tumors may have a growth advantage compared to their DAPK-negative counterparts [17]. In contrast, Mittag et al. [18] describe DAPK promoter hypermethylation as a very early event in colorectal carcinogenesis with a high frequency in T1 tumors [18]. In inflammation-associated colorectal carcinogenesis, DAPK seems to play an important role in tumor transformation [19]. Taken together, all these reports support an antagonistic duality for DAPK dependent on the cellular context and the different experimental settings [20].

Although DAPK is involved in a variety of cellular functions such as cell death, migration, and invasion, so far *in vivo* studies do not identify DAPK expression levels in different tumor regions such as the tumor center and the tumor invasion front. Nevertheless, both regions differ remarkably in regard to the number of tumor infiltrating immune cells such as T-lymphocytes, macrophages, or dendritic cells [21, 22]. The tumor microenvironment and tumor-host-interaction at the invasion front has been identified as having prognostic value in colorectal cancer [21]. We have shown previously that in colorectal cancer there is a cross-talk between tumor and immune cells mainly at the invasion front [23]. Here tumor-associated macrophages influence the tumor border gene expression pattern [24] and DAPK-mediated pro-apoptotic responses [25]. Moreover, so-called tumor buds detach from the tumor as single cells or small cell clusters (up to five cells) and are also scattered in the stroma at the invasion front. Colorectal cancers with high-grade tumor budding very often show an infiltrative diffuse growth pattern associated with advanced tumor stage and poor clinical outcome. Interestingly, tumor buds display very low proliferation rates [26], an increased migratory capacity [27] and have been linked to an epithelial-mesenchymal transition (EMT) [28]. Disseminating tumor buds are known to down-regulate pro-apoptotic molecules such as apoptosis activating factor 1 (APAF1) [29] and only rarely express Caspase-3 [26] to protect themselves from anoikis, a form of cell death by cell detachment.

So far, DAPK has never been investigated in these aggressive tumor cells. Since high-grade tumor budding has been associated with metastasis, we aimed to study their DAPK expression and to link it to functional properties of tumor aggressiveness *in vitro*.

## RESULTS

### DAPK expression was decreased towards the invasion front and was nearly absent in disseminating tumor buds

**Figure 1:**
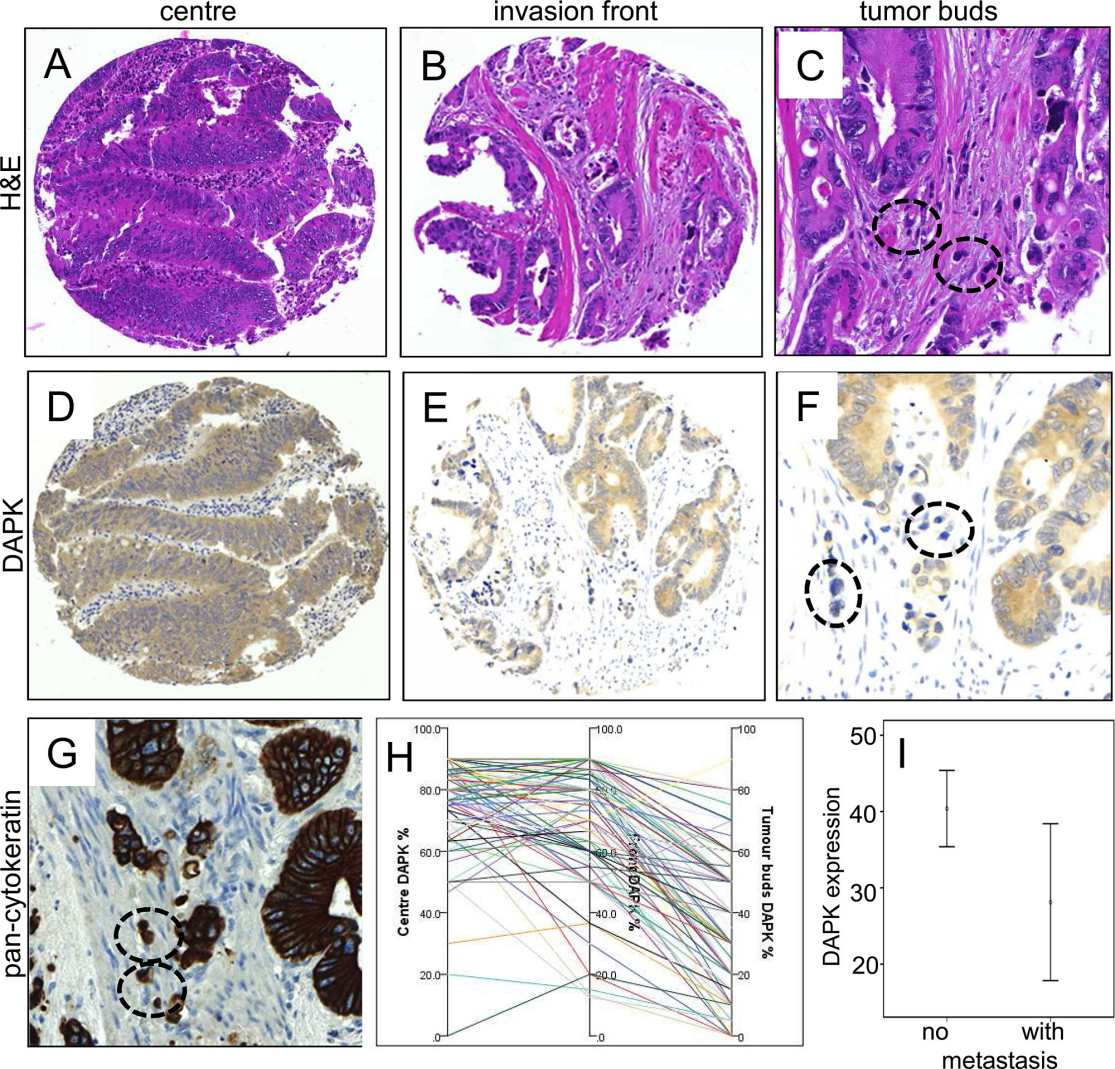
Immunohistochemical staining of DAPK protein expression in tumors of colorectal cancer patients Examples of tumor center **A, D.** and invasion front **B, E.** in comparison with H&E staining (A, B) and DAPK immunohistochemistry (D, E). Overall there is only a faint reduction of staining intensity from tumor center to the invasion front. The detailed analyzes of tumor buds in H&E **C.,** a standard pan-cytokeratin staining (AE1-3, routine protocol, **G.** and DAPK immunohistochemistry **F.** revealed an almost vanishing DAPK expression in these small tumor cell clusters (encircled). A summary of the DAPK gradients across these tumor areas in the same samples is plotted in **H.** Of note, the tumor bud pictures highlight the interaction with dense inflammatory infiltrates (C, F, G). **I.** Statistical analysis of DAPK expression in tumor buds of patients with or without metastasis (*p* = 0.034).

DAPK was evaluated in a TMA containing 144 colorectal cancers and was found in the cytoplasm of the tumor cells (Figure 1). In comparison to the tumor center (76.4% expression), DAPK expression was reduced towards the invasion front (72.1% expression) (*p* = 0.0352) and was nearly lost in tumor buds (38.6% expression) (*p* < 0.0001, Figure 1F, 1H; Table 1). Expression of DAPK in tumor center, front and within tumor buds was not correlated with clinicopathological features (Table 2). Although DAPK in the center did not correlate with any prognostic features, mucinous cancers showed less DAPK at the invasion front (*p* = 0.0445). Most importantly, a significant loss of DAPK in tumor buds was associated with presence of distant metastasis (*p* = 0.0291, Figure 1A). There was, however, no association of DAPK expression with survival time.

**Table 1:** Descriptive statistics for DAPK expression in the tumor center, invasion front and tumor buds

DAPK	No. cases	Mean (%) ± SD	Median (%)	Min (%)	Max (%)
Tumor center	144	76.4 ± 15.5	80	20	90
Invasion front	134	72.1 ± 19.6	80	12.5	90
Tumor buds	91	38.6 ± 21.8	40	0	90
Difference center – invasion front	134	4.39 ± 18.2	0	−30	70
Difference front – tumor buds*	89	33.0 ± 19.4	31.6	−20	90

* significant loss of DAPK expression toward invasion front (*p* = 0.0352) and from front to tumor buds (*p* < 0.0001)

**Table 2:** Association between DAPK expression (percent positivity) in tumor center, front and tumor buds with clinicopathological features

Feature		Center (*n* = 144)		*P*-value	Front (*n* = 134)		*P*-value	Buds (*n* = 91)		*P*-value
		*N*	Mean ± SD		*N*	Mean ± SD		*N*	Mean ± SD	
Gender (*n* = 144)	Male	66	72.2 ± 18.9	0.0334	62	74.1 ± 18.5	0.2751	42	39.7 ± 22.1	0.63
	Female	78	79.9 ± 10.7		72	70.3 ± 20.5		49	37.6 ± 21.7	
										
Patient age (*n* = 144) (years)	Corr. coeff	144	−0.073	0.3871	134	−0.14	0.1061	91	−0.035	0.736
										
Tumor size (*n* = 144) (cm)	Corr. coeff	144	−0.036	0.6654	134	−0.077	0.3752	91	−0.007	0.945
										
Histological subtype (*n* = 144)	Other	130	75.7 ± 16.0	0.1649	121	73.0 ± 19.5	0.0445	81	38.5 ± 21.4	0.939
	Mucinous	14	82.8 ± 7.0		13	62.9 ± 18.1		10	39.5 ± 25.9	
										
Tumor grade (*n* = 144)	G1–2	97	75.4 ± 15.8	0.2106	90	74.3 ± 16.7	0.3123	63	38.9 ± 19.8	0.8896
	G3	47	78.4 ± 14.9		44	67.4 ± 24.0		28	37.9 ± 25.9	
										
Tumor location (*n* = 144)	Left	87	76.8 ± 14.7	0.6438	82	73.9 ± 18.8	0.3217	52	38.2 ± 20.1	0.9777
	Rectum	18	71.0 ± 21.2		18	68.0 ± 19.5		13	38.1 ± 23.8	
	Right	39	77.9 ± 13.9		34	69.6 ± 21.4		26	39.6 ± 24.6	
										
pT classification (*n* = 144)	pT1–2	38	77.5 ± 14.8	0.6978	36	71.9 ± 19.2	0.7806	24	34.8 ± 18.4	0.3177
	pT3–4	106	76.0 ± 15.7		98	72.1 ± 19.8		67	39.9 ± 22.8	
										
pN classification (*n* = 144)	pN0	78	74.5 ± 17.6	0.495	75	72.4 ± 18.6	0.7753	50	37.7 ± 20.7	0.5982
	pN1–2	66	78.6 ± 12.3		59	71.6 ± 20.9		41	39.6 ± 23.3	
										
pM classification (*n* = 144)	pM0	125	76.8 ± 15.5	0.1818	117	72.7 ± 19.3	0.2707	75	40.8 ± 21.7	0.0291
	pM1	19	73.8 ± 15.4		17	67.4 ± 21.6		16	28.1 ± 19.3	
										
Venous invasion (*n* = 144)	Present	27	76.2 ± 14.3	0.6017	25	71.1 ± 21.5	0.8807	18	36.4 ± 25.0	0.6367
	Negative	117	76.4 ± 15.8		109	72.3 ± 19.2		73	39.1 ± 21.1	
										
Lymphatic invasion (*n* = 144)	Present	57	78.5 ± 12.8	0.4896	51	71.7 ± 21.3	0.5847	39	39.1 ± 24.3	0.7802
	Negative	87	75.0 ± 17.0		83	72.3 ± 18.5		52	38.2 ± 19.9	
										
Post-operative therapy (*n* = 144)	None	57	76.1 ± 15.1	0.8771	55	75.5 ± 20.1	0.6674	34	34.4 ± 19.8	0.1828
	Treated	87	76.5 ± 15.8		79	71.7 ± 19.3		57	41.1 ± 22.7	
										
Tumor budding (10-in-10) (*n* = 144)	Low-grade	78	75.1 ± 15.5	0.1876	71	75.3 ± 17.3	0.0894	39	46.3 ± 17.5	0.0024
	High-grade	66	77.9 ± 15.4		63	68.5 ± 21.4		52	32.8 ± 23.0	
										
Survival (*n* = 144)	HR (95%CI)	144	0.998 (0.983–1.014)	0.7983	134	0.991 (0.978–1.004)	0.1805	91	0.996 (0.981–1.011)	0.5771

### DAPK loss elevates tumor cell random migration

**Figure 2:**
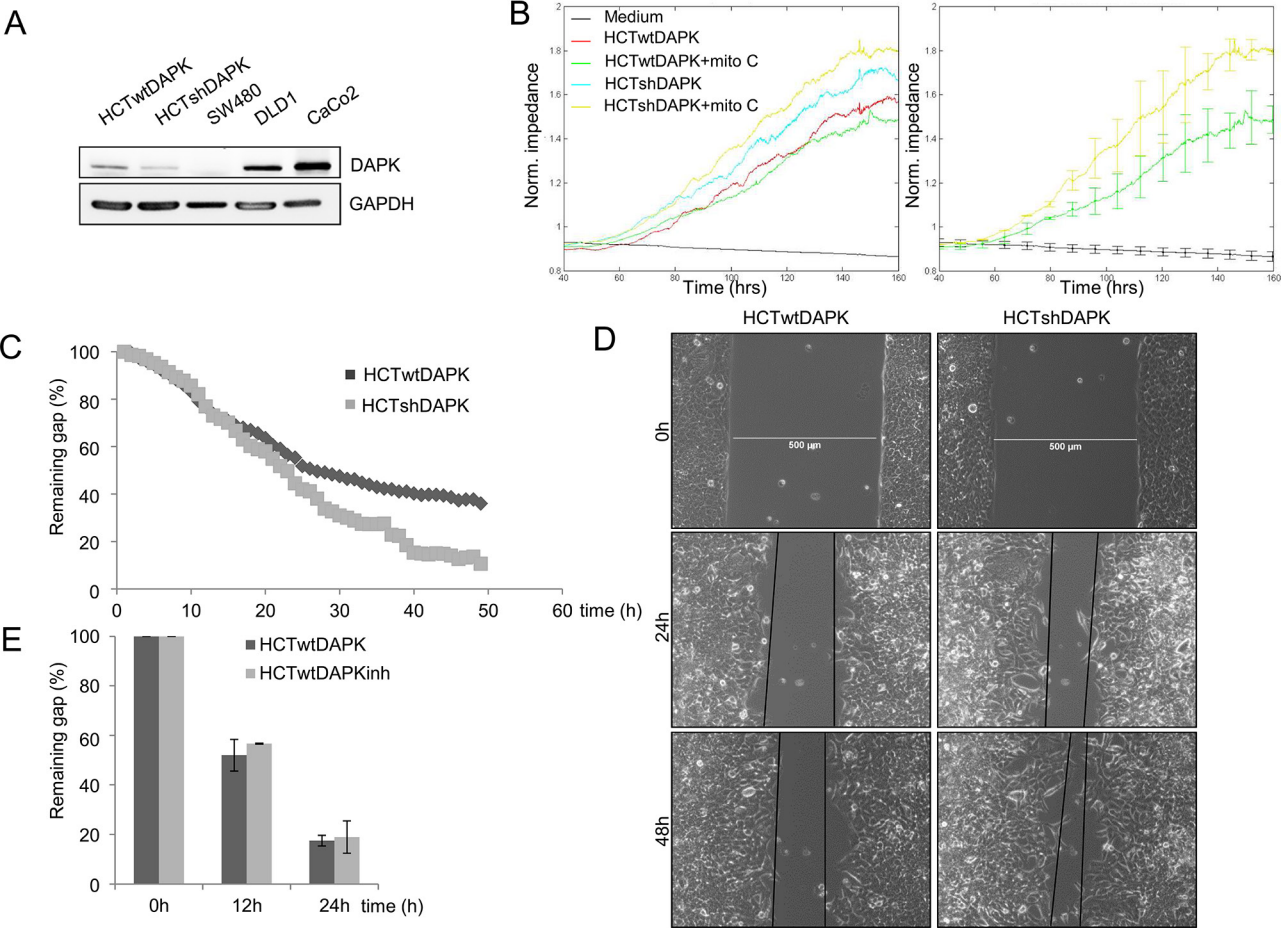
Negative effect of DAPK on random and wound healing migration **A.** DAPK expression in colorectal cancer cells lines. Cell lysates were subjected to immunoblot analyzes with antibodies as indicated. Anti-DAPK (BD Transduction Laboratories) and anti-phospho DAPKSer308 (Sigma), with secondary antibodies (anti-mouse IgG peroxidase conjugated; Pierce). Bound antibodies were visualised using West Pico chemiluminescent substrate (Pierce) according to the manufacturer's instructions. Immunoblotting with anti-GAPDH was used to control equal loading and protein quality. Images were captured using GeneGnome (Syngene). **B.** Electrical cell impedance sensing (ECIS) traces for HCTshDAPK and HCTwtDAPK cells with or without mitomycin C (10 ng/ml) treatment over time. Experiment was repeated three times. Representative graphs are shown. **C.** Quantification of the results describes the change in percentage of the wound size at the indicated times for wound healing migration monitored by live-cell imaging microscopy. **D.** Bright-field imaging in wound healing migration assay for HCTshDAPK and HCTwtDAPK cells. Cell migration into wound monitored by live-cell imaging microscopy and bright-field images were captured at the indicated times after wounding. Bar, 500 μm. **E.** A bar graph showing the number of cells that migrated after the treatment with DAPK inhibitor. Data are represented as means ± SD from two independent experiments. 500 μm wound was set as 100% remaining gap.

Owing to the previous proven *in vivo* reduction of DAPK, we performed several *in vitro* assays to study the role of DAPK in tumor cell aggressiveness in colorectal cancer cells to understand how DAPK affects cellular functions in disseminating tumor cells. As a first step we determined the level of DAPK expression in different colorectal tumor cell lines to select the suitable *in vitro* model. Low DAPK protein levels were observed in the HCTshDAPK and SW480 cells whereas DAPK was expressed moderately in HCT116, DLD1, and Caco2 cells (Figure 2A). Next, we utilised the ECIS technology to examine the effects of DAPK on random cell migration. If cells attach, migrate and spread on the small gold film electrode of the ECIS array, cellular membranes restrict the electrical current, forcing it to flow beneath and between the cells, resulting in a dramatic increase in electrical impedance. We discovered an increase in electrical impedance for HCTshDAPK cells suggesting that migration was elevated when DAPK was lost (Figure 2B). Because proliferation was inhibited using mitomycin C (10 ng/ml, Sigma) this seems to indicate a clear migratory effect (Figure 2B). In addition, the proliferation rate for both cell lines was independent of the DAPK status (Supplementary Figure S2).

### DAPK expression decreases the migratory capacity in wound healing assay

**Figure 3:**
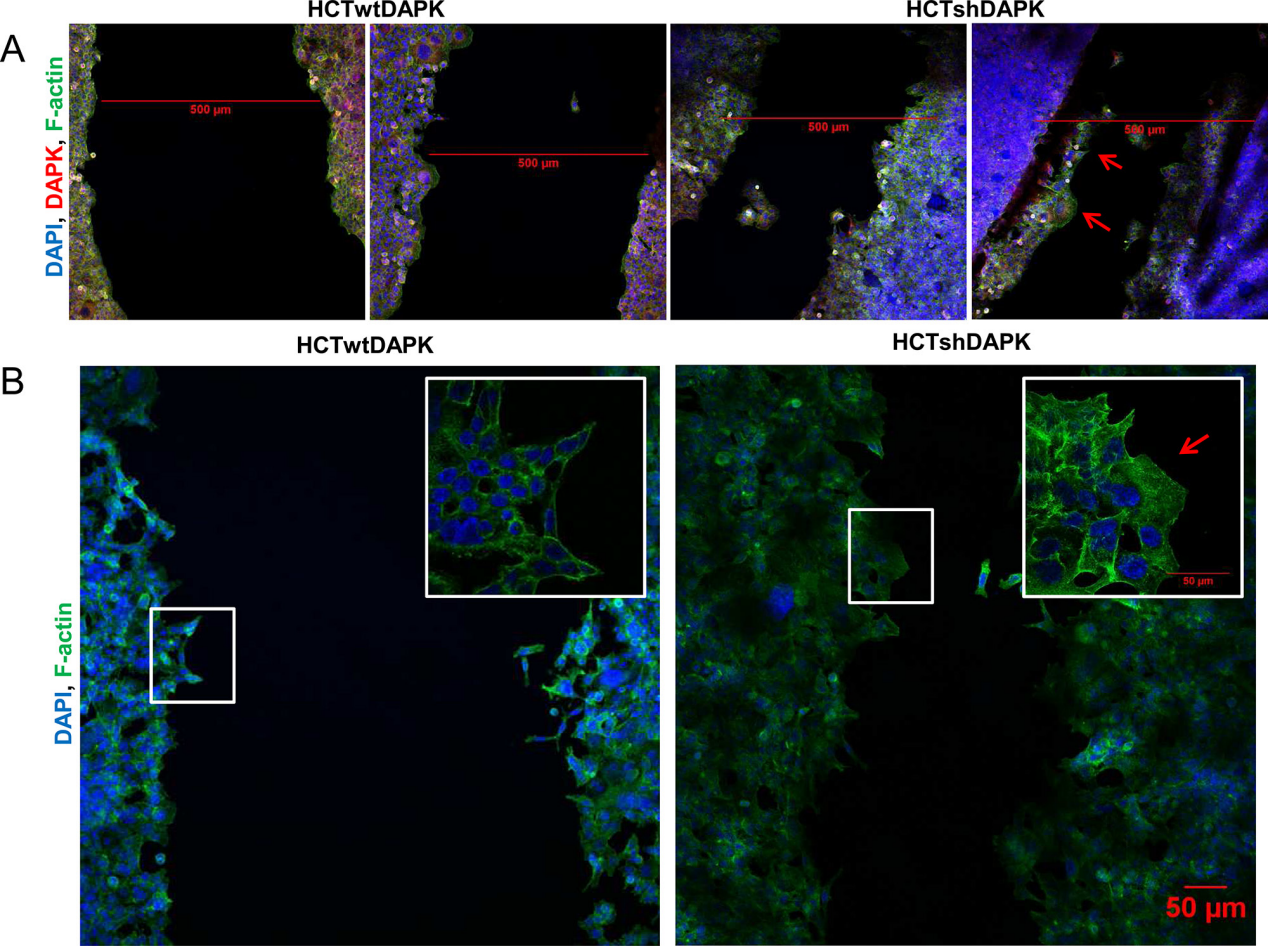
Confocal immunofluorescence images in wound healing migration assay for HCTshDAPK and HCTwtDAPK cells **A.** HCTshDAPK and HCTwtDAPK cells were grown for 24–48 h allowing migration under mitomycin C (10 ng/ml) treatment, fixed and stained with the endogenous DAPK(red), F-actin (green) and DAPI (blue). Bar, 500 μm. **B.** HCTshDAPK and HCTwtDAPK cells were cultured for 24 h–48 h allowing migration under mitomycin C (10 ng/ml) treatment. Cells were fixed and stained with phalloidin for F actin (green) and DAPI (blue). Confocal immunofluorescence images were taken Bar, 50 μm.

In the second experiment, wound healing migration assay of HCTwtDAPK and HCTshDAPK cells with culture inserts was performed and cell migration into the wounded area was monitored and measured by live-cell imaging microscopy (Figure 2C, 2D). After 24 h and 48 h, HCTshDAPK cells showed accelerated cell migration into the wounded area, whereas wound closure of HCTwtDAPK cells was reduced, showing a larger remaining gap (Figure 2C, 2D), (Supplementary Movie film S1/S2). An almost completed monolayer was formed by HCTshDAPK cells within 96 h (Supplementary Figure S3). Treatment with DAPK inhibitor did not affect cell migration (Figure 2E). Interestingly, there was a DAPK-dependent difference in migration pattern. Whilst the HCTwtDAPK cells showed mostly single cells migrating into the wound, HCTshDAPK cells showed a rather cluster-like pattern of larger cell groups migrating (Figure 3A). Immunofluorescence staining showed DAPK-dependent differences in the F-actin reorganisation at the wound margin. In HCTwtDAPK cells F-actin distribution was rather cortical, demonstrating less-polarised and less-migrating cells. In contrast, HCTshDAPK cells showed polarized lamellipodia formation of F-actin, typical for migrating cells with a clear orientation of the tumor cell nuclei opposite the wound margin (Figure 3B).

**Figure 4:**
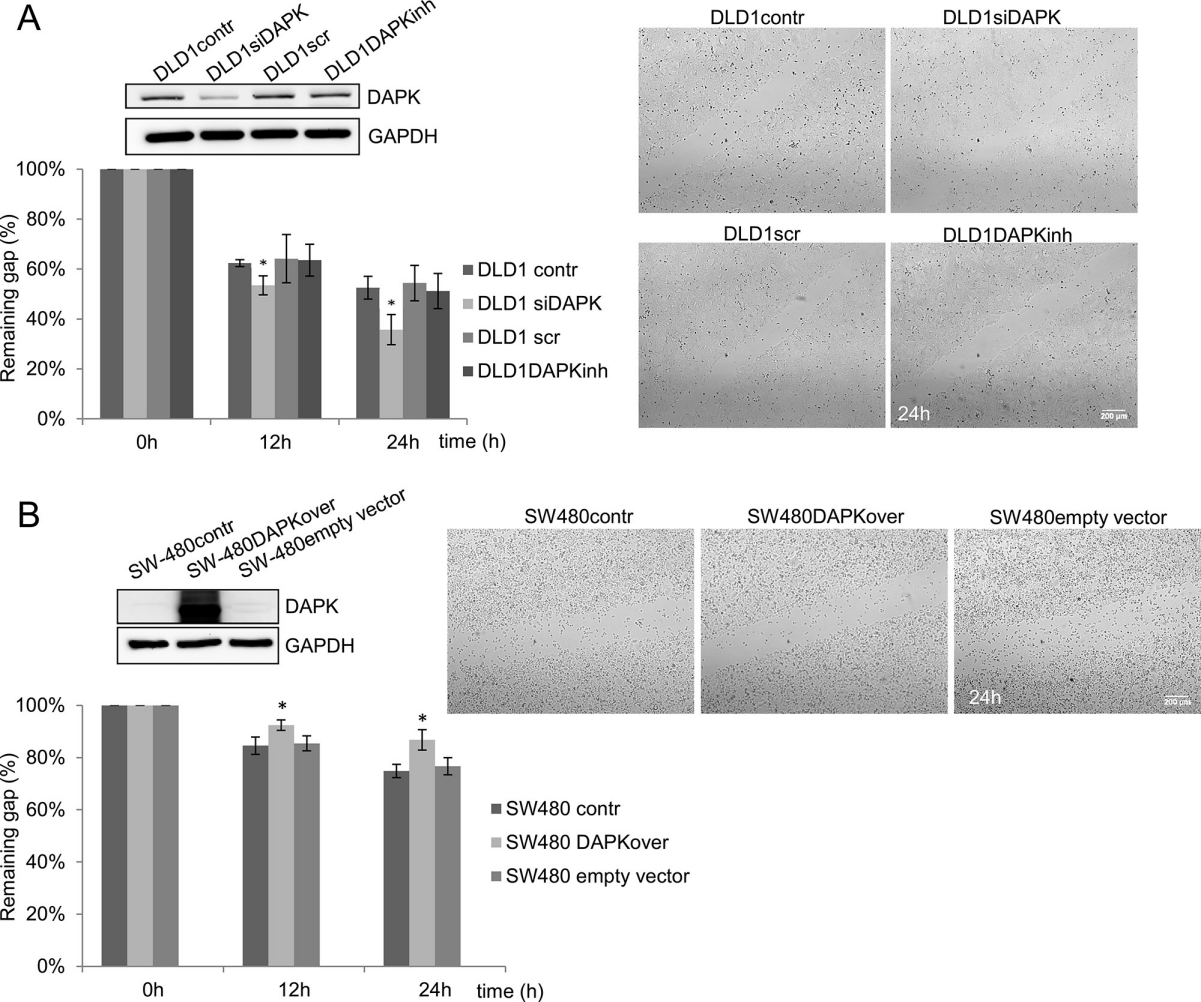
DAPK level is a determining factor in tumor cell migration **A.** DAPK siRNA transfection in DLD1 cells; effect of scratch wound healing after gene silencing. **B.** Transfection of SW480 with DAPK vector; effect of scratch wound healing after gene overexpression. Quantification of the results describes the change in percentage of the wound size at the indicated times. Data shown represent means ± SD (*n* = 3). 500 μm wound was set as 100% remaining gap. Bar, 200 μm. *p < 0.05 for DAPK expression modulation compared with control.

Next, we examined the effect of DAPK loss in DLD1 cells. DAPK knockdown cells demonstrated an increase in cell migration in comparison to DLD1 control cells (Figure 4A) showing a significantly reduced percentage of the remaining gap for DAPK siRNA treated cells 12 h and 24 h after the scratch was created. The inhibition of DAPK catalytic activity in DLD1 cells did not reveal any change in the migration rate (Figure 4A).

In SW480 cells with low endogenous DAPK expression we used the contrary strategy and investigated the effects of overexpressed DAPK on migration. Indeed, DAPK overexpression decreased cell migration 12 h and 24 h after the scratch was created. DAPK overexpressing cells demonstrated a significantly higher percentage of remaining gap in comparison to SW480 control cells (Figure 4B).

Taken together, these results show that DAPK plays an important role in decreasing the migratory capacity and substrate phosphorylation by DAPK would not appear to be crucial for this effect.

### DAPK negative cells have a higher invasive potential but do less tolerate anchorage independence conditions

**Figure 5:**
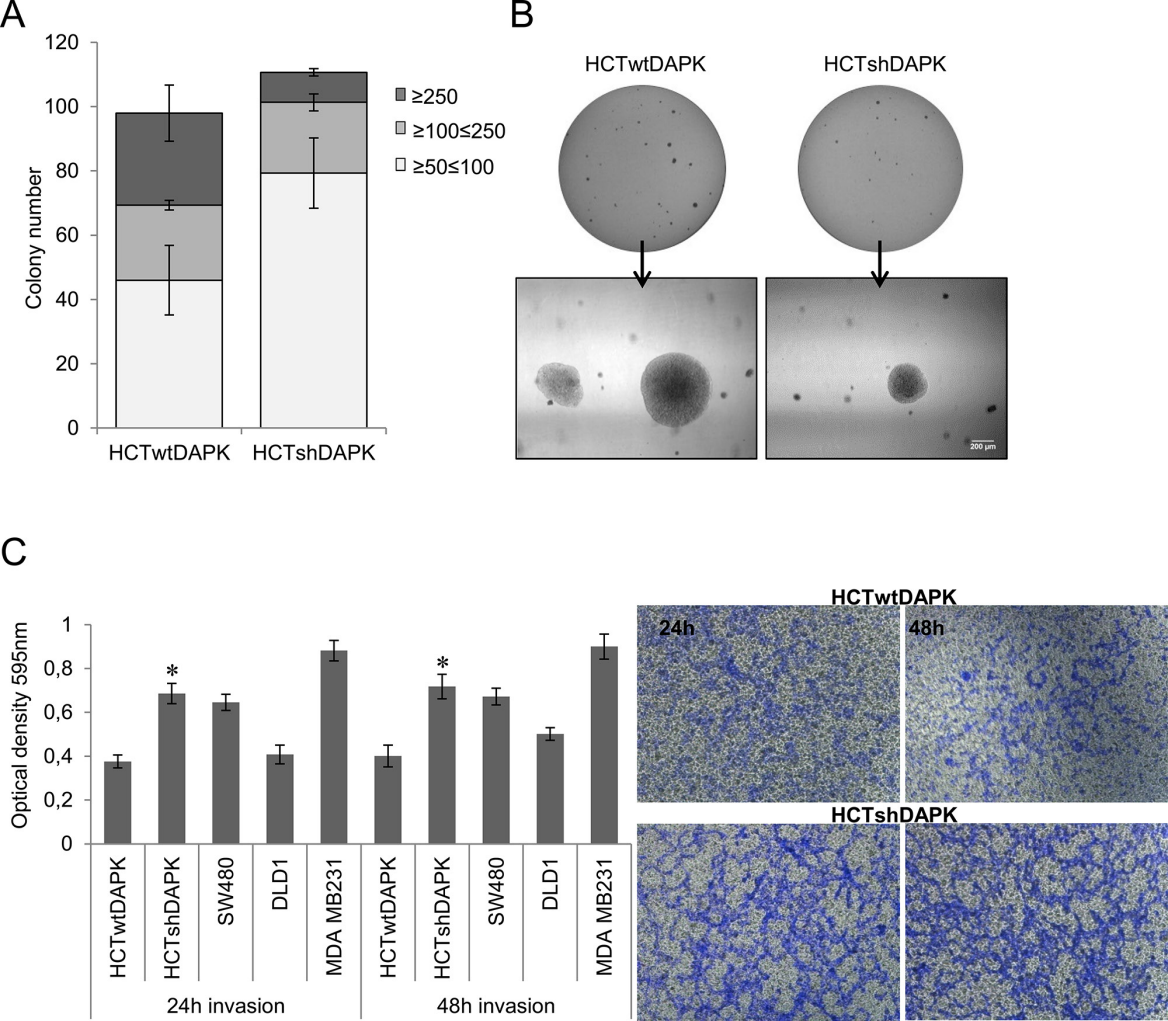
Colony formation and invasion assay of HCTshDAPK and HCTwtDAPK cells **A.** Colonies were fixed and stained with crystal violet (0.5%), photographed and counted visually **B.** Bar, 200 μm. Values are based on different size of colony diameter from four different fields. Data shown represent means ± SD (*n* = 2). **C.** Transwell invasion assay was performed using the Corning Costar Matrigel Matrix coated migration chamber (8 μm pore size). Cells were seeded in the upper chamber in medium with 5% fetal bovine serum, and left for the invasion through the matrigel membrane for 24 h and 48 h into the lower chamber well with 10% fetal bovine serum medium. The upper surface of the filter was swabbed with cotton to remove non-invading cells. Invading cells were stained with 0.5%(w/v) crystal violet, quantified by solubilising bound crystal violet in 1%(w/v) sodium dodecyl sulfate (SDS) for 1 hour at 37°C. Absorbance was measured at 595 nm; *p < 0.05 compared with control; data represent mean ± SD from two independent experiments with two replicates. The photos of invading cells through the Matrigel membrane stained with crystal violet were taken in separated fields at 100× total magnification.

To study the effect of endogenous DAPK protein level on anchorage independent growth in HCTwtDAPK and HCTshDAPK cells we performed the soft agar colony formation assay. We observed that the number of colonies overall (≥50 μm, ≥100 μm, ≥250 μm) did not differ for the two cell lines (Figure 5A, 5B). Whilst HCTshDAPK cells showed a higher number of smaller colonies (50–100 μm), more than 50% of HCTwtDAPK colonies were larger than 100 μM in size. Since the proliferation rate for both cell lines was independent of the DAPK status (Supplementary Figure S2) these data are consistent with the assumption that DAPK loss induces cell cycle perturbations. In the subsequent experiment, we examined the effect of DAPK on cell invasion by using Boyden chambers coated with Matrigel. We compared the invasion characteristics of different colorectal tumor cell lines using the human mammary carcinoma cell line MDA-MB-231 as a positive control. The invasion potential of HCTshDAPK cells was increased 1.8-fold compared with parental HCTwtDAPK cells (Figure 5C, Supplementary Figure S4, and Supplementary Figure S5).

## DISCUSSION

Recently, DAPK has been shown to exert pro- and anti-inflammatory as well as pro- and anti-apoptotic functions in a context- and stimulus-dependent manner [30]. The invasion front of a colorectal tumor is the region of tumor-immune cell interaction where inflammation and tumor cell dissemination occurs. There are only a few reports about the molecular mechanisms by which factors at the tumor invasion front alter the gene expression profile of neighbouring epithelial cells. Indeed, there seems to be a panel of signalling molecules differently expressed in the tumor center and the invasion front [31]. Kahlert et al. [32] show an invasion-front specific pattern of miRNAs when compared to normal tissue. In contrast, using laser capture microdissection another study by Staub et al. [33] describes rather similar gene expression profiles at the invasion front and the inner tumor mass where the normal epithelium differs remarkably from the tumor compartments. Despite a similar transcriptome, the authors suggest that posttranscriptional mechanisms in response to tumor and immune cell interaction such as protein stabilisation may be much more affected. It is known that extracellular proteins of the tumor microenvironment induce genes at the invasion front of colorectal liver metastases that are associated with epithelial-mesenchymal transition (EMT), a process that is a crucial step for tumor dissemination [32]. Indeed, the stability of these EMT-inducing transcription factors is remarkably regulated by the proteasome system [34].

Our *in vivo* results suggested that immunohistochemical DAPK protein expression was not associated with clinico-pathological parameters and patient outcome. Nevertheless, there is a close agreement with Chen et al. [15] who reported that DAPK down-regulation was associated with metastasis to lymph nodes and distant organs, as well as with a shorter metastasis-free period. We found that the M1 status was significantly correlated with low DAPK level in tumor buds.

Immunohistochemical studies by our group and others underline the similarities between tumor buds and the epithelial-mesenchymal transition (EMT). In particular, tumor buds lose E-cadherin from the tumor cell membrane and over-express markers of EMT, anoikis-resistance, invasion and migration [27]. Despite the clear associations between tumor budding and a more aggressive phenotype, little is known about the molecular profile of these disseminating aggressive cells.

To understand the functional role of DAPK at the tumor invasion front or in the tumor buds we used *in vitro* models of established colorectal tumor cell lines. Interestingly, colony formation assay showed that colorectal tumor cells with or without DAPK form the same number of colonies, whereby DAPK expressing cells develop a higher number of larger colonies. Since these differences cannot be attributed to different proliferation rates, the delay in colony formation for DAPK low or non-expressing cells may reflect the induction of an early cell cycle arrest or a disturbance in cell cycle progression. In this regard, it has been described that the invasion front of colorectal carcinomas are zones of low proliferation [35]. Here, it was demonstrated that a cell cycle inhibitor such as p16 was upregulated at the invasion front and its increased expression at the infiltrative front of invasion of human colorectal cancer was a prognostic marker for poor survival [35, 36]. Since the invasion front represents a cancer site where early steps of tumor cell dissemination exist, we wanted to explore the role of DAPK loss in migration. During directed migration, DAPK functions as a potent inhibitor of cell polarisation, whereas DAPK inhibits random migration by suppressing directional persistence [6]. The DAPK's motility-inhibitory function is thought to have two significant functions in suppressing tumor development and/or progression. Firstly, as cell migration is necessary for tumor invasion through the basement membrane, DAPK may suppress metastasis through its effect on cell migration, thereby acting in the late stage of tumor progression [6]. Secondly, the motility–inhibitory effect of DAPK would be important to suppress tumors that have escaped from DAPK-induced apoptosis [6]. Indeed, by using Boyden chamber assay, we demonstrated that DAPK knockdown in colorectal cancer cells resulted in increased cell invasion.

We can only speculate how DAPK is regulated in tumor buds and at the invasion front and for this we need to consider the role of chronic inflammation in carcinogenesis and the function of immune cells expressing inflammatory cytokines [37, 38]. Tumor cells are recognised by the immune system. Active immune escape mechanisms have therefore been considered a novel hallmark of cancer [39]. Thus far almost nothing is known about DAPK's role at the invasion front of a colorectal tumor representing a dynamic interface between pro- and anti-tumor factors. The infiltrating tumor border configuration and tumor budding promote progression and dissemination of tumor cells by penetrating the vascular and lymphatic vessels [21]. We showed that in Barrett's carcinomas the tumor-associated macrophages express DAPK protein and they are rarely detectable in tumors with DAPK hypermethylation, thereby suggesting a possible cross-talk between immune and tumor cells [40]. In recent study from our group, using a cell culture model with conditioned supernatants of differentiated/activated macrophages (U937) and human HCT116 colorectal tumor cells, we simulated immune cell mediated DAPK-associated tumor cell death, reflecting the *in vivo* tumor setting. Indeed, supernatants of freshly isolated human macrophages were able to induce DAPK expression [25]. Therefore, DAPK expression at the invasion front could be regulated by inflammatory pathways or by modulations of the tumor microenvironment. In this regard we identified a new function of DAPK in suppressing TNF-induced activation of the pro-inflammatory transcription factor STAT3 [19]. Thus the DAPK loss in tumor buds is well fitting with the observation that STAT3 is known to be upregulated at the invasion front of squamous cell carcinoma [41].

We suggest that DAPK down regulation at the invasion front and in tumor buds could represent a novel immune escape mechanism that requires further study. A better understanding of tumor heterogeneity will help to define new therapeutic strategies to overcome therapy resistance.

## MATERIALS AND METHODS

### Patients

A retrospective cohort of 144 patients with primary colorectal cancer treated at the Fourth Department of Surgery, University of Athens Medical School in Athens, Greece, between 2002 and 2007 participated in this study. For each patient, a complete histopathological review was undertaken; follow-up, survival time and treatment information were available from patient charts. None of the patients received neoadjuvant therapy. The following information was available: gender, age, tumor size, TNM stage (6^th^ edition), tumor grade, presence of venous and lymphatic invasion, and tumor location (Table 3). Median survival time for the cohort was 60 months. The clinical endpoint of interest was overall survival. The use of patient material and data was approved by the ethics committee of the University of Athens. In order to ensure that the patient cohort was representative, Kaplan-Meier survival curves were plotted for overall survival, followed by tumor grade, pT, pN, and pM. All showed the expected survival differences (*p* < 0.001, Supplementary Figure S1). Tumor budding was scored as low-grade or high-grade according to the 10-in-10 method [42]. A considerable and negative prognostic effect of high-grade budding on survival (*p* < 0.001) was observed.

**Table 3:** Patient characteristics (*n* = 144)

Feature		Freq (%)
Gender (*n* = 144)	Male	66 (45.8)
	Female	78 (54.2)
Patient age (*n* = 144) (years)	Mean ± SD	69.4 ± 11.5
		
Tumor size (*n* = 144) (cm)	Mean ± SD	4.6 ± 2.0
		
Histological subtype (*n* = 144)	Other	130 (90.3)
	Mucinous	14 (9.7)
		
Tumor grade (*n* = 144)	G1–2	97 (67.4)
	G3	47 (32.6)
		
Tumor location (*n* = 144)	Left	87 (60.4)
	Rectum	18 (12.5)
	Right	39 (27.1)
		
pT classification (*n* = 144)	pT1–2	38 (26.4)
	pT3–4	106 (73.6)
		
pN classification (*n* = 144)	pN0	78 (54.2)
	pN1–2	66 (45.8)
		
pM classification (*n* = 144)	pM0	125 (86.8)
	pM1	19 (13.2)
		
Venous invasion (*n* = 144)	Present	27 (18.8)
	Negative	117 (81.2)
		
Lymphatic invasion (*n* = 144)	Present	57 (39.6)
	Negative	87 (60.4)
		
Post-operative therapy (*n* = 144)	None	57 (39.6)
	Treated	87 (60.4)
		
Tumor budding (10-in-10) (*n* = 144)	Low-grade	78 (54.2)
	High-grade	66 (45.8)
		
Survival (*n* = 144)	5-year survival rate % (95%CI)	42.5 (26–58)

### Tissue microarray and immunohistochemistry (IHC)

A next-generation tissue microarray (ngTMA) with 0.6 mm tumor cores was constructed [43] and included three spots of tumor center, three of invasion front and two spots of the densest tumor budding regions, where present. The TMA was then sectioned at 3 μm and immunohistochemistry was performed with a Leica Bond III system (Leica Biosystems, Buffalo Grove, IL, USA) in addition to a manual overnight incubation with primary antibody anti-DAPK (1:50) (BD Transduction Laboratories, Lexington NY) at 37°C. After washing with buffer (Dako), sections were incubated with the secondary antibody at RT for 30 min. Secondary antibodies were EnVision+System HRP linked (goat-anti-mouse or goat-anti-rabbit, Dako) and positive immunoreactivity was detected using DAB+ (Dako) chromogen substrate. Nuclei were counterstained with haematoxylin (Dako). Appropriate positive and negative controls were included in each run of IHC. The percentage of tumor cells that stained positive (immunoreactivity above the background, percentage of positive cells 10–90%) was quantified/scored by an experienced pathologist (TR). Then the average expression across the punches was calculated. A separate score for the tumor center, invasion front and tumor buds were given.

### Statistics

Using a signed-rank test for matched pairs, differences in DAPK expression between tumor regions was carried out. A non-parametric test (Wilcoxon's Rank Sum or Kruskal Wallis test) was used to test associations of DAPK expression in tumor center, invasion front and tumor buds with clinicopathological features, while a Pearson's correlation coefficient was used to describe the strength of the linear relationship between age, size and DAPK expression. Cox regression analysis with hazard ratio and 95%CI was used to detect survival time differences with DAPK, while the Kaplan-Meier method and log-rank test were applied for categorical data. *P*-values < 0.05 were considered statistically significant. Analyzes were performed using SAS V9.2 (The SAS Institute, Cary NC, USA).

### Cell culture and transfection

Human colorectal HCT116, Caco 2, DLD1 and SW480 tumor cells were maintained in RPMI and human mammary carcinoma cell line MDA-MB-231 was maintained in DMEM supplemented with 10% fetal bovine serum, penicillin (100 U/ml), and streptomycin (100 μg/ml). HCT116 DAPK shRNA stable cell line was generated using DAPK shRNA lentiviral particles according to the manufacturer's instructions (Santa Cruz), as previously described [44]. All cell lines were maintained at 37°C in a humidified atmosphere and 5% CO_2_. Mycoplasma contamination was excluded by using the PCR based system. Cell lines were authenticated using Multiplex Cell Authentication by Multiplexion (Heidelberg, Germany) as described recently [45]. As DAPK1 inhibitor we used (4Z)-2-phenyl-4-(pyridin-3-ylmethylidene)-4,5-dihydro-1,3-oxazol-5-one (MolPort). Cells were pre-treated for 60 min with 25 μM DAPK inhibitor as previously published [5]. Transient transfection was performed using Invitrogen's lipofectamin 2000 according to the manufacturer's recommendations as previously described [4, 19].

### Western blotting

Equal protein content (30 μg) of cell lysates were separated on denaturing SDS-PAGE and transferred onto a nitrocellulose membrane prior to probing with antibodies as indicated and described previously [4, 19].

### Assessment of cell migration by electric cell substrate impedance sensing (ECIS)

The effect of endogenous DAPK protein expression on cell migration was analyzed applying the ECIS system. The cell lines (HCTwtDAPK and HCTshDAPK) were seeded in a density of 7500 cells per well on 8W10E ECIS arrays (an 8-well chamber slide device - each well consisting of 10 active working planar gold film electrodes) and grown to confluence over 2 − 5 days. To prevent proliferation, the cells were treated with mitomycin C (10 ng/ml, Sigma). The eight-well electrode array was placed in a humidified cell culture incubator at 37°C with 5% CO_2_ and connected to the electronic devices located outside the incubator. The difference in the impedance of the two cell lines with different DAPK levels was analyzed by comparing the average of normalised impedance. The changes in electrical resistance were monitored in real time using an ECIS Z (Theta) instrument (Applied Bio-Physics) with 8W10E ECIS arrays and results expressed as impedance [46, 47].

### Wound healing migration assay

A wound healing migration assay was used to measure basic cell migration parameters such as speed, persistence, and polarity. 3 × 10^5^ cells were seeded in a confluent layer with culture inserts (Ibidi, Martinsried, Germany) and incubated in RPMI medium. After 24 h, cells were treated with mitomycin C (10 ng/ml, Sigma) and inserts were removed with sterile forceps to create a wound field of ~500 μm. Additionally, cells were seeded in a 6-well plate at a density of 3 × 10^5^cells/well for DAPK siRNA transfection, DAPK overexpression, and DAPK inhibitor treatment. After the monolayer confluence was reached, a straight wound or scratch was then gently created in the cell monolayers with a sterile pipette tip. Cells detached during the scratch were washed with PBS and cultures were then supplemented with the fresh medium containing mitomycin C (10 ng/ml, Sigma). The cells were incubated and monitored for an additional 24 h-72 h at 37°C. Bright field microscopy images were taken with an inverted microscope Nikon eclipse Ti-U (Tokyo, Japan) using 4×/20× objective lens (Nikon). For the live cell imaging microscopy, phase contrast images were collected at 10 minutes intervals over 60 hours using a Keyence BZ9000 microscope, with a 20× objective.

Fixed cells were incubated with blocking buffer 1%BSA in PBS and then immunofluorescence staining was performed with anti-DAPK (1:250) (BD Transduction Laboratories, Lexington NY) and F-actin using Alexa Fluor 350-conjugated plalloidin (Invitrogen) at 1:10 (incubated for 30 min at room temperature). Alexa Fluo 555-conjugated secondary antibody (Invitrogen) was used at a concentration 1:500. Cells were mounted on slides with ProLong Gold antifade reagent with DAPI (Invitrogen). Immunofluorescence images were acquired with Confocal Laser Scanning Microscopy system (LSM T-PMT Observer Z1, Carl Zeiss Inc.), using a 63x oil objective.

### Soft agar assay for colony formation

Colony formation assay on soft agar [48] was performed to determine the effect of DAPK expression level on anchorage independent growth in HCTwtDAPK and HCTshDAPK cells. To prepare the base layer, 0.6% agarose in RPMI media was added to 6-well plates and allowed to polymerise. The top layer was prepared by producing 0.3% agar in cell culture media, and following cooling to approximately 40°C, 2000 cells were mixed in 2 ml of top layer agar and plated over the base layer. Plates were allowed to solidify and then incubated at 37°C. After 18 days, when colonies reached a size which were microscopically clearly visible, colonies were counted and images were taken. The cells were stained and fixed with 0.5% crystal violet in 20% methanol solution. The experiment was repeated in duplicate.

### Invasion assay

For invasion assay, 5 × 10^4^ cells were seeded on the top of Transwell membranes (8 μm pore size) coated with Matrigel basement membrane matrix (Corning Costar) following the manufacturer's instructions. After 24 h and 48 h of incubation, filters were fixed with 3% paraformaldehyde (15 min) and stained with 0.5%(w/v) crystal violet, quantified by solubilising bound crystal violet in 1%(w/v) sodium dodecyl sulfate (SDS) for 1 hour at 37°C. Absorbance was measured at 595 nm.

### Cell proliferation assay

Cell Proliferation Kit II XTT (Roche Biodiagnostics GmbH, Penzberg, Germany) and Cell Counting kit −8 (Dojindo Molecular Technologies, Inc. Rockville, MD 20850, USA) were used according to the manufacturer's instructions to detect cell proliferation. For the growth curve assay cells were counted with trypan blue and counted at each time point.

## SUPPLEMENTARY FIGURES AND MOVIE






