# Correction: DAPK loss in colon cancer tumor buds: implications for migration capacity of disseminating tumor cells

**DOI:** 10.18632/oncotarget.16154

**Published:** 2017-03-13

**Authors:** Jelena Ivanovska, Inti Zlobec, Stefan Forster, Eva Karamitopoulou, Heather Dawson, Viktor Hendrik Koelzer, Abbas Agaimy, Fabian Garreis, Stephan Söder, William Laqua, Alessandro Lugli, Arndt Hartmann, Tilman T. Rau, Regine Schneider-Stock

**Present**: Due to an error in proofreading, Figures 2 and 4B were incorrectly identified.

**Figure 2:**
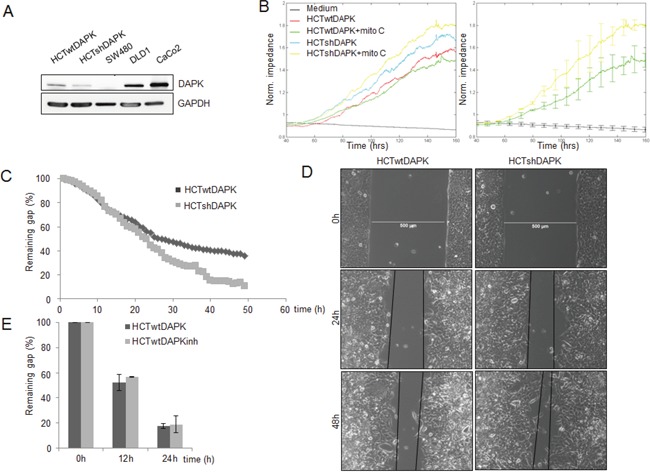
Negative effect of DAPK on random and wound healing migration A. DAPK expression in colorectal cancer cells lines. Cell lysates were subjected to immunoblot analyzes with antibodies as indicated. Anti-DAPK (BD Transduction Laboratories) and anti-phospho DAPKSer308 (Sigma), with secondary antibodies (anti-mouse IgG peroxidase conjugated; Pierce). Bound antibodies were visualised using West Pico chemiluminescent substrate (Pierce) according to the manufacturer's instructions. Immunoblotting with anti-GAPDH was used to control equal loading and protein quality. Images were captured using GeneGnome (Syngene). B. Electrical cell impedance sensing (ECIS) traces for HCTshDAPK and HCTwtDAPK cells with or without mitomycin C (10 ng/ml) treatment over time. Experiment was repeated three times. Representative graphs are shown. C. Quantification of the results describes the change in percentage of the wound size at the indicated times for wound healing migration monitored by live-cell imaging microscopy. D. Bright-field imaging in wound healing migration assay for HCTshDAPK and HCTwtDAPK cells. Cell migration into wound monitored by live-cell imaging microscopy and bright-field images were captured at the indicated times after wounding. Bar, 500 μm. E. A bar graph showing the number of cells that migrated after the treatment with DAPK inhibitor. Data are represented as means ± SD from two independent experiments.500 μm wound was set as 100% remaining gap.

**Figure 4:**
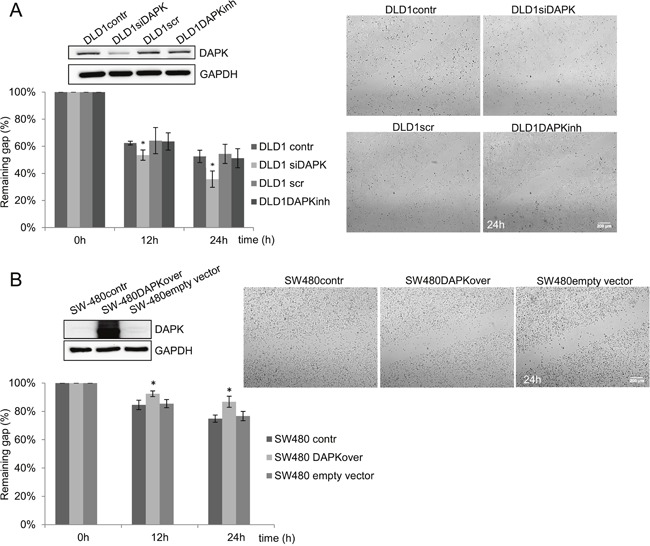
DAPK level is a determining factor in tumor cell migration. **A.** DAPK siRNA transfection in DLD1 cells; effect of scratch wound healing after gene silencing. **B.** Transfection of SW480 with DAPK vector; effect of scratch wound healing after gene overexpression. Quantification of the results describes the change in percentage of the wound size at the indicated times. Data shown represent means ± SD (*n* = 3). 500 μm wound was set as 100% remaining gap. Bar, 200 μm. *p < 0.05 for DAPK expression modulation compared with control.

**Correct**: The proper figures are given below. The author sincerely apologizes for this error.

Original article: Oncotarget. 2015; 6(34):36774-88. doi: 10.18632/oncotarget.4908.

