# Supplementary material for: DAPK loss in colon cancer tumor buds: implications for migration capacity of disseminating tumor cells
Source: Oncotarget. 2015 Aug 21;6(34):36774–88. doi: 10.18632/oncotarget.4908 (PMC4742210; doi:10.18632/oncotarget.4908)
Supplement: Supplementary file 1 [file oncotarget-06-36774-s001.pdf]

SUPPLEMENTARY FIGURES AND MOVIE

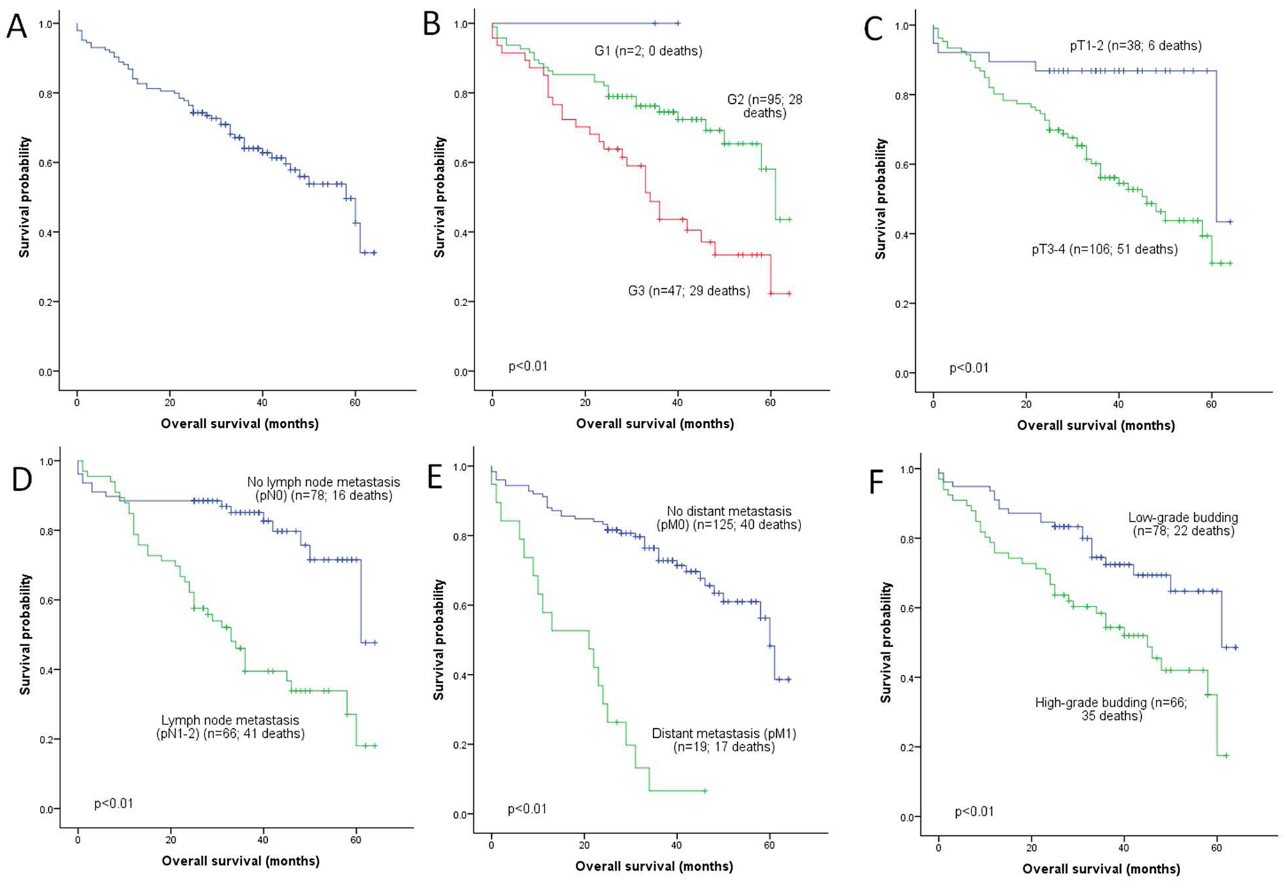

Supplementary Figure S1: Standard features of prognosis. Kaplan-Meier survival curves highlighting. **A.** overall survival of the entire cohort, worse overall survival with **B.** higher tumor grade, **C.** more advanced pT classification, **D.** lymph node metastasis (pN1) compared to no lymph node metastasis (pN0), **E.** distant metastasis (pM1) compared to no distant metastasis (pM0) and finally **F.** with high-grade tumor budding compared to low-grade budding. Log-rank test.

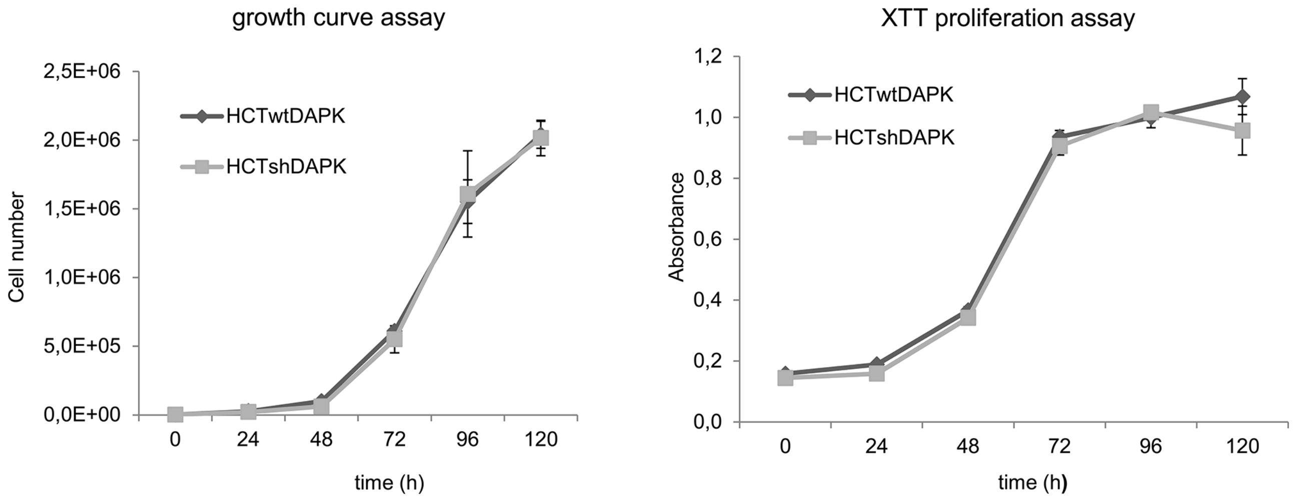

Supplementary Figure S2: Growth curve and cell proliferation analyzes for HCTshDAPK and HCTwtDAPK cells. For the growth curve analyzes, cells were counted with trypan blue and counted at each time point. Data shown represent means ± SD ($n = 3$).

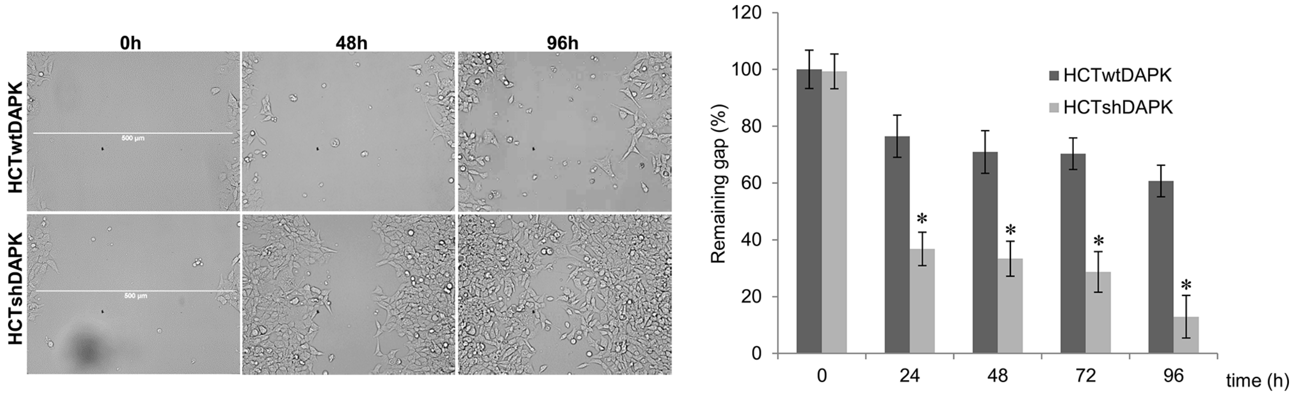

Supplementary Figure S3: Bright-field images were captured at the indicated times after wounding for HCTshDAPK and HCTwtDAPK cells. HCTshDAPK and HCTwtDAPK cells were grown for 96 h allowing migration under mitomycin C (10 ng/ml) treatment. Bar, 500 µm. Quantification of the results describes the change in percentage of the wound size at the indicated times for wound healing migration. 500 µm wound was set as 100% remaining gap. Data shown represent means ± SD ($n = 2$); *$p < 0.05$ compared with control.

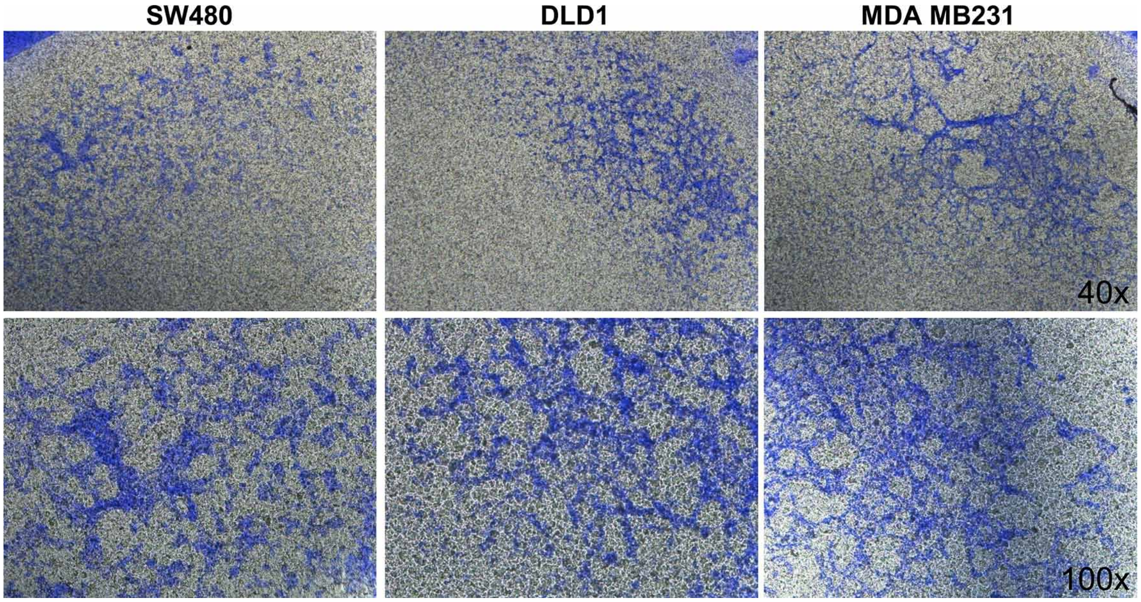

Supplementary Figure S4: Invasion assay for colorectal cancer cells lines and human mammary carcinoma cell line MDA-MB-231 used as a positive control in invasion. Transwell invasion assay was performed using the Corning Costar Matrigel Matrix coated migration chamber (8 µm pore size). The invading cells through the matrigel membrane were stained with 0.5%(w/v) crystal violet and photos were taken in separated fields at 40× and 100× total magnification 24 h post invasion.

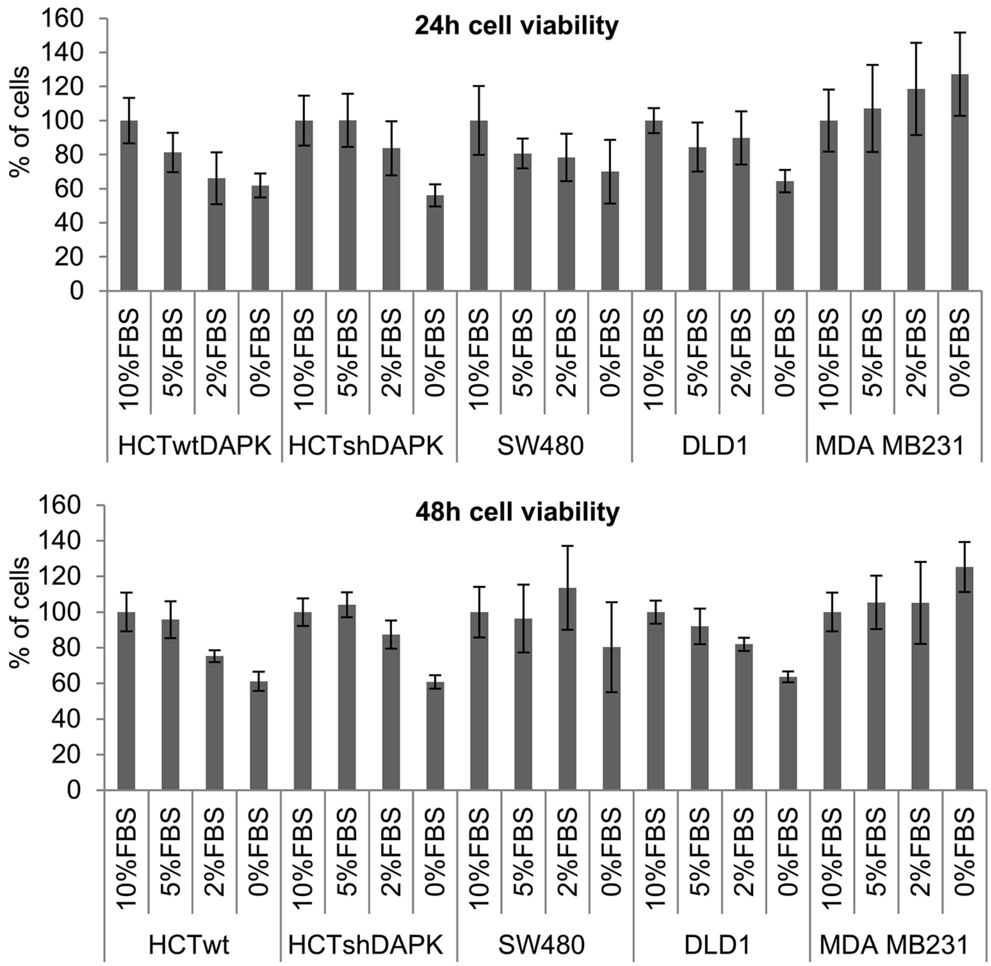

Supplementary Figure S5: Cell viability measurement for the cell lines used in transwell invasion assay cultured with different percentage of fetal bovine serum in medium. Human colorectal DLD1 and SW480 tumor cells and human mammary carcinoma cell line MDA-MB-231(used as a positive control for the invasion) were seeded with different concentration of fetal bovine serum (10%, 5%, 2% and 0%) and cultured for 24 h or 48 h. Cell viability was measured at indicated times in order to define the lowest concentration of fetal bovine serum that could be used for viable cells in the upper well during the invasion assay.

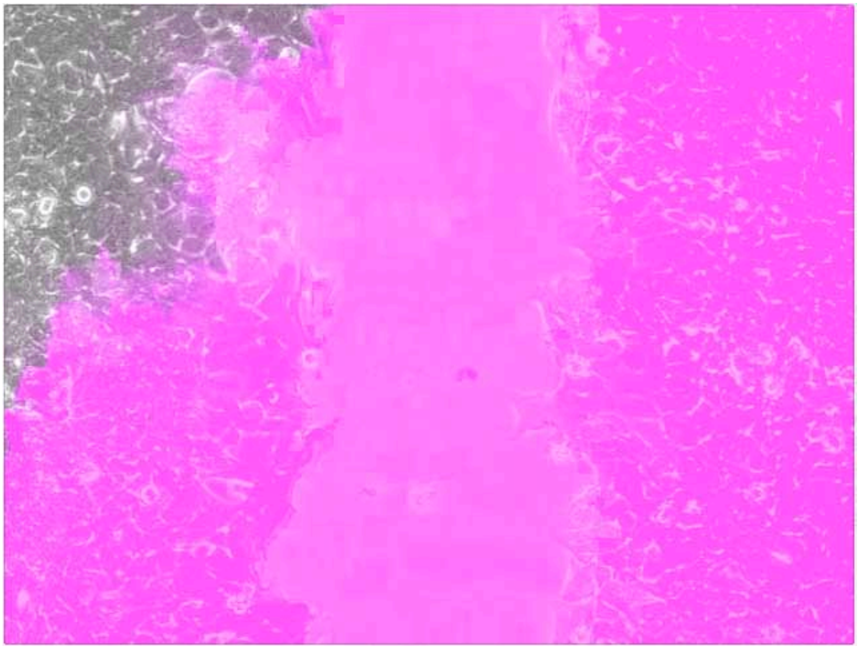

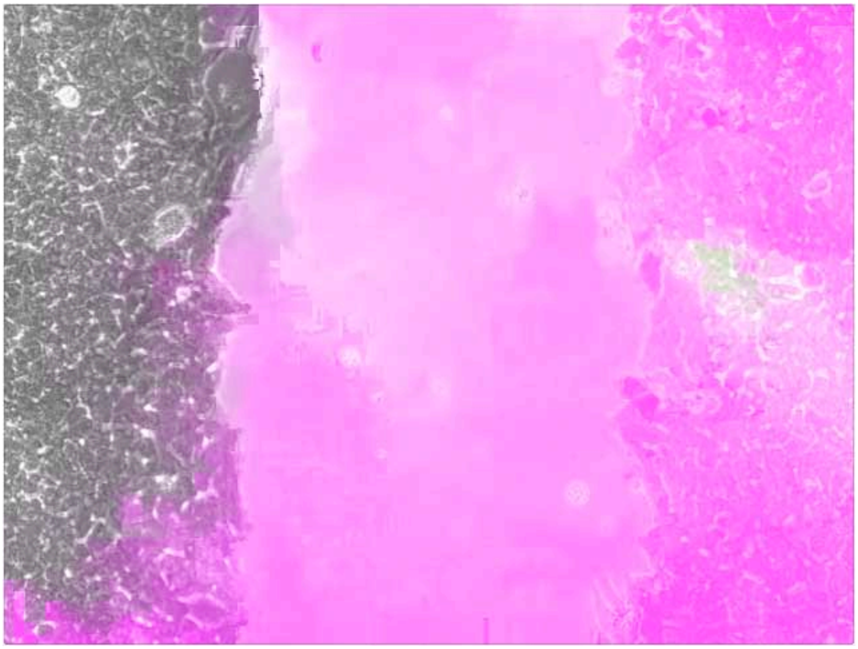

Supplementary Movie File S1, S2: Migration into wound for HCTwtDAPK (Supplementary Movie File S1) and HCTshDAPK (Supplementary Movie File S2) cells under mitomycin C (10 ng/ml) treatment monitored by live-cell imaging microscopy. Phase contrast images were collected 10 minutes intervals over 60 hours with a 20× objective lens.
